# Aging Behavior of High-Viscosity Modified Asphalt Binder Based on Infrared Spectrum Test

**DOI:** 10.3390/ma15082778

**Published:** 2022-04-10

**Authors:** Wenxuan Zhang, Qiang Li, Jiaqing Wang, Yuanpeng Meng, Zhou Zhou

**Affiliations:** 1 College of Civil Engineering, Nanjing Forestry University, Nanjing 210037, China; zhangwenxuan@njfu.edu.cn (W.Z.); liqiang2526@njfu.edu.cn (Q.L.); myp564335@njfu.edu.cn (Y.M.); 2 School of Transportation, Southeast University, Nanjing 211189, China; zhouzhou1024@seu.edu.cn

**Keywords:** porous asphalt pavement, high-viscosity modified asphalt, functional group index, aging kinetics

## Abstract

In the rapid development of sponge city construction in China, porous asphalt pavement has been widely used. The high-viscosity modified asphalt used for porous asphalt pavements is utilised in a complex aging environment. In this study, infrared spectroscopy was used to test the changes in the functional groups of high-viscosity modified asphalt under the influence of ultraviolet radiation intensity, high temperature, and water corrosion conditions. The research results showed that under the influence of several environmental factors, the high-viscosity modified asphalt has no chemical reaction but does undergo physical changes. From the perspective of the functional group index, the carbonyl index is more suitable for evaluating the degree of ultraviolet aging, and the sulfoxide group index is more suitable for evaluating the effect of temperature on aging. The high-viscosity modified asphalt aging kinetic models, established with different functional group indexes as indicators, have different activation energies. The aging kinetic model established with the carbonyl index is more suitable for simulating traditional thermal-oxidative aging. This study provides a better plan to reveal the influence of different environmental factors on the aging performance of high-viscosity modified asphalt under complex environmental conditions.

## 1. Introduction

Within the concept of building a green resource-saving society, “sponge cities” have attracted more and more attention from relevant government departments and practitioners. Building porous asphalt pavements is one of the effective ways to promote the construction of sponge city infrastructure [1]. Asphalt pavement with large voids has been widely used in roads due to its advantages of anti-slip ability, drainage, and noise reduction. At the same time, the main asphalt binder used in porous asphalt is high-viscosity modified asphalt (HVA), which is excessively exposed to oxygen, temperature, water, and ultraviolet radiation, thereby accelerating its aging damage, which leads to the deterioration of asphalt road performance [2,3,4]. The polymer degradation and polymer phase structure destruction of high-viscosity modified asphalt is distinct from that of conventional modified asphalt, which will cause some differences in performance between high-viscosity modified asphalt and conventional modified asphalt, especially in aging performance [5,6,7].

At present, the research focus of high-viscosity modified asphalt is mainly concentrated on preparation, modification mechanism, pavement performance, and so on. Luo [8] et al. studied the performance of the modified mixture with different high-viscosity agents, and the results showed that the most appropriate content of the high-viscosity modifier is 12% of the asphalt mass. Cai [6] et al. prepared environmental-friendly HVA using waste materials and studied the formation mechanism of its physical and mechanical properties through a series of microscopic experiments. However, the research on high-viscosity modified asphalt is still insufficient, especially that of its aging performance, which is very important to extending the durability of porous asphalt pavement.

The influence of environmental factors on the aging behavior of high-viscosity modified asphalt has also attracted the attention of researchers. Li [9] et al. studied the aging performance of HVA under complex environmental conditions. The results show that acid rain solution and ultraviolet light also have a significant impact on viscoelasticity, high and low temperature performance, and fatigue performance. It is proposed that the coupling aging effect should be considered during service. Yang [10] studied the thermal aging behavior of HVA from the perspective of cohesion, adhesion, and rheology, and the results showed that aging weakened the cohesion, adhesion, and low-temperature performance of HVA, and reduced its surface roughness. Hu [11] discussed the aging characteristics of high-viscosity modified asphalt under accelerated weathering conditions and analyzed the effects of temperature and aging time on its rheological properties and chemical changes. Li [12] studied the influence of different ultraviolet radiation intensities on asphalt aging and used DSR and FTIR tests to study the changes in rheological properties. The results showed that different ranges of ultraviolet light have different degrees of influence on asphalt aging, among which 360 nm ultraviolet light has the most significant impact on asphalt. Zou [13] analyzed the influence of different acid-base environments on asphalt aging at 60 °C, and the study showed that acid and alkaline solutions have more significant effects on asphalt aging. The current research mainly focuses on the impact of single environmental factors on the aging of high-viscosity modified asphalt. The actual service environment of high-viscosity modified asphalt is more complicated and is affected by multiple environmental factors. The aging effect is not yet clear. It is necessary to couple high-viscosity modified asphalt under different factors research on aging behavior.

Infrared spectroscopy is used as a rapid detection method to observe the changes in asphalt microstructure. It can qualitatively analyze and study the structure and chemical composition changes of asphalt cement before and after aging from a microscopic point of view, and it can also quantitatively characterize asphalt cement before and after aging [14,15]. Yang et al. [16] used FTIR to characterize the rheological properties and aging mechanism of asphalt binders mixed with high percentages of bio-adhesives. The analysis showed that the effects of aging bio-adhesives stem from three aspects: the loss of volatiles, dehydrogenation of high molecular weight compounds such as asphaltenes, and oxidation of chemical substances. Yao et al. [17] used FTIR to test different asphalt binders to obtain the spectra of chemical groups. They found that carboxylic acids and ketones are the main aging components of asphalt when exposed to air and oxygen. In addition, these functional groups and carbonyl groups are closely related to the rutting sensitivity and fatigue of asphalt mixtures. Bhupendra [18] used infrared spectroscopy to study the aging characteristics of conventional asphalt, polymer-modified asphalt (PMB), and warm-mix asphalt (WMA), and quantified the obtained infrared spectroscopy results by calculating different indicators, finally calculating the aging index of short-term and long-term aging of asphalt based on these indicators.

Infrared spectroscopy can quickly identify and mark the functional groups in asphalt to judge its chemical composition. Based on Lambert Beer law, it can carry out absolute quantitative analysis with high precision [15]. Most researchers currently use infrared spectroscopy to study and quantify the effect of aging on asphalt binders, but there are few studies on the aging characteristics of high-viscosity modified asphalt using infrared spectroscopy. As for the selection of environmental factors, some scholars choose a single UV, or UV and temperature coupled environmental conditions. However, porous asphalt pavement is always exposed to complex environments, including sunlight, ultraviolet light, and rainfall [9]. Therefore, this article is based on infrared spectroscopy experiments to study the aging characteristics of high-viscosity modified asphalt under multi-factor coupled environmental conditions, discusses the influence of different environmental factors on the chemical components of high-viscosity modified asphalt, and finally establishes the high-viscosity modified asphalt aging kinetic equation based on the functional group index. This study shows that it is of great significance to consider the coupling effect of different environmental factors on the aging of high-viscosity modified asphalt, which is helpful to better evaluate the on-site aging behavior of porous asphalt pavement.

## 2. Materials and Methods

### 2.1. Materials

In this paper, SBS modified asphalt provided by Jiangyin Taifu Company (Jiangyin, China) was selected as the base asphalt, in which the content of SBS was 4.5%. TPS (TAFPACK-Super) high-viscosity agent was added to the SBS for preparation of high-viscosity modified asphalt (HVA), which was prepared in accordance with the Chinese standard JTGT 3350-03-2020. TPS modifier is a high-viscosity modifier specially produced for porous asphalt pavement. The full name of TPS is TAFPACK-Super. Its composition is mainly thermoplastic rubber, supplemented by adhesive resin, combined with other stabilizers to make ordinary asphalt become high-viscosity modified asphalt through mechanical mixing and processing. The TPS high-viscosity agent was in 12 wt.% of the base binder. SBS asphalt and TPS high-viscosity agent were put into a high-speed shearing machine, shearing for 30 min at 180 °C and 5000 rpm. Asphalt samples with a diameter of 140 mm and a thickness of about 0.7–1.0 mm were prepared after mixing, then settled in the asphalt aging simulation device for aging procedures. The 0.7–1.0 mm asphalt sample was prepared by pouring, and the purpose of controlling the thickness of asphalt film was achieved by controlling the mass of the pouring volume. The asphalt was heated to 180 °C, and then poured into the regular glass dish with a consistent diameter. The bottom surface of the glass dish was a circle with a radius of 7 cm, where the area was determined. After measuring the density and quality of the asphalt sample, the thickness of the asphalt sample thickness can be controlled by the pouring volume. The physical properties of base bitumen (SBS) and HVA are shown in Table 1, and the physical properties of the high-viscosity agent are shown in Table 2.

### 2.2. Complex Aging Simulation Method

The ultraviolet radiation intensity in China can be divided into three gradients: 35–50 × 10^3^ J/cm^2^ a, 25–35 × 10^3^ J/cm^2^ a, and 20–25 × 10^3^ J/cm^2^ a; the three selected ultraviolet high-pressure mercury lamp radiation intensity levels represent the national ultraviolet distribution gradient: 10.14 × 10^−4^ w/cm^2^, 13.28 × 10^−4^ w/cm^2^, 17.38 × 10^−4^ w/cm^2^; the water situation of acidity, neutrality, and alkalinity represents the effects to high-viscosity modified asphalt by moisture in the service process. The alkaline solution was prepared by diluting pure NaOH reagent with a mass fraction of 7%, and pH = 11 was determined by pH meter. The acid solution was made of sulfuric acid and nitric acid by selecting the ratio of C (SO_4_^2−^): C (NO_3_^−^) = 9:1 to simulate the acid rain solution, and determined the pH = 2.8. In the conventional aging simulation method, PAV used three temperature levels of 90 °C, 100 °C, and 110 °C. To ensure the authenticity and aging efficiency of the simulation, three temperature levels of 90 °C, 100 °C, and 110 °C were selected to simulate thermal aging.

**Figure 1:**
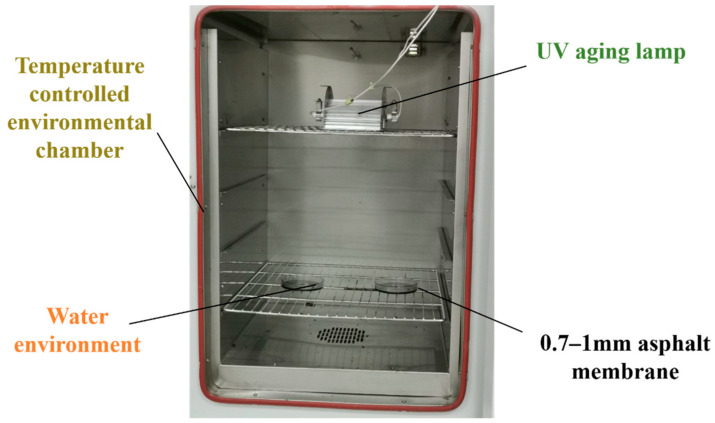
Complex aging simulation device.

This article adopts a self-designed coupling aging device, which can combine three environmental factors to achieve simultaneous effects of ultraviolet light, temperature, and moisture. The simulated aging equipment is composed of an environmental box, an ultraviolet high-pressure mercury lamp, and a water-immersed transparent vessel, as shown in Figure 1. The device can adjust the distance between the lamp and the test piece via the card slot to change the ultraviolet irradiance on the surface of the test piece, control the temperature through the environmental box to simulate thermal aging, and use the forming of a uniform water film on the surface of the asphalt sample to simulate water aging. It also changes the solution every five hours to ensure the integrity of the water film and the purity of the solution during the aging process.

### 2.3. FTIR Spectroscopy Test

A non-destructive FTIR test was performed to detect the changes in functional groups of asphalt under the influence of different environmental factors. First, a small amount of potassium bromide was ground into a powder to produce a compressed sample. Then, the heated high-viscosity pitch was evenly coated on the pressed samples of potassium bromide. Cary 630 infrared spectrometer was used to collect data in the attenuated total reflection (ATR) mode. Its wave number accuracy was greater than 0.005, the signal-to-noise ratio was greater than 5000, and 32 times per scan. The samples were heated to 70 °C, then a few samples were evenly coated on all the surfaces of ATR crystals, and their infrared spectra were measured.

### 2.4. Experiment Plan

The aging test plan is shown in Table 3, and the sample aging time was 40 h. “O-HVA” stands for origin HVA; “U” stands for HVA aging under the condition of ultraviolet radiation intensity; “UT” means HVA aging under the combined action of ultraviolet light and high temperature; and “UTS” means HVA aging under the combination of ultraviolet light, temperature, and different water quality solutions. At the same time, different factor levels were embedded in the coupling conditions. L, M, and H mean low, medium, and high level, respectively, A indicates acidic water conditions, and ALK indicates alkaline water condition. For example, “U_L_T_L_S_N_” represents low-level ultraviolet irradiance, low-level temperature, and neutral water quality conditions.

## 3. Results

### 3.1. The Effect of UV Intensity on Aging

The functional groups corresponding to the special absorption peaks of the infrared spectrum are shown in Table 4. The quantitative analysis of infrared spectroscopy is based on the peak area ratio related to the characteristic functional groups in the asphalt. The definition of the peak area ratio is as follows [15].

Aliphatic functional group index:

(1) $$I_{B}=A_{1377}/\left(A_{1462}+A_{1377}+A_{723}\right)$$

Aromatic functional group index:

(2) $$I_{Ar}=\frac{A_{1600}}{\sum^{\;}A}$$

Carbonyl functional group index:

(3) $$I_{C=O}=\frac{A_{1700}}{\sum^{\;}A}$$

Sulfoxide functional group index:

(4) $$I_{S=O}=\frac{A_{1031}}{\sum^{\;}A}$$

where: 

*A*—Different peak areas; $\sum^{\;}A$—The sum of different peak areas in a certain range. $\;\sum^{\;}A=A_{1700}+A_{1600}+A_{1456}+A_{1376}+A_{1306}$ + $A_{1162}$ + $A_{1031}$ + $A_{968}$ + $A_{861}$ + $A_{810}$ + $A_{744}$ + $A_{722}$.

**Figure 2:**
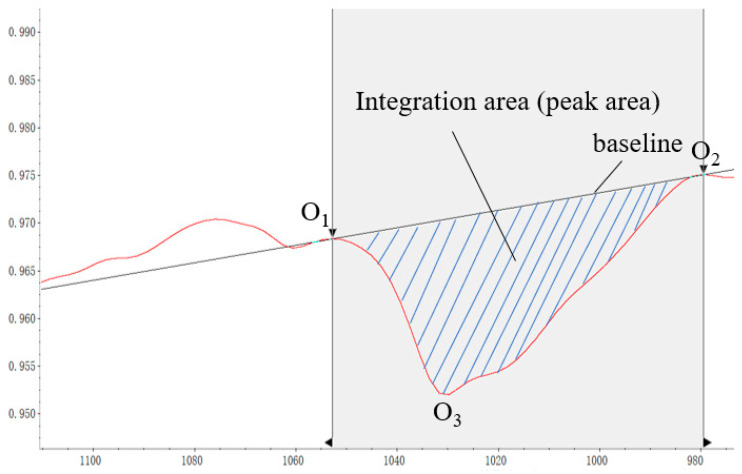
Peak area calculation diagram.

The spectrum analysis software adopts OMNIC, takes the sulfoxide group at 1031 cm^−1^ of the original HVA as an example, and takes the tangent of the lowest point on both sides of the absorption peak as the correction baseline from which to calculate the absorption peak area *A*_1031_. The schematic diagram of peak area calculation and baseline selection is shown in the Figure 2 (O_1_, O_2_, and O_3_ are the reference points for peak area calculation). Similarly, different peak areas corresponding to other functional groups can be obtained, and the indexes of different functional groups can be calculated according to the above Formulas (1)–(4).

**Figure 3:**
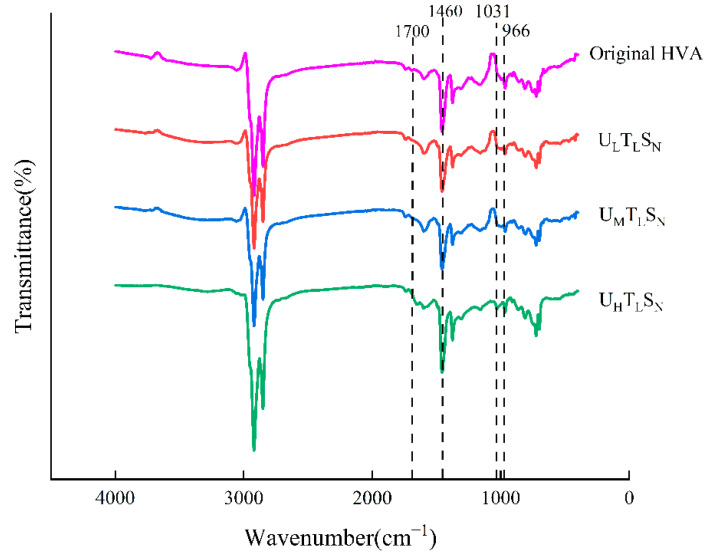
Variation of the spectrum of different asphalt samples under different ultraviolet irradiation intensities.

The infrared spectra of asphalt samples under different levels of ultraviolet radiation are shown in Figure 3. Compared with the original HVA, after three levels of ultraviolet radiation, the intensity of the carbonyl group at 1700 cm^−1^ and the sulfoxide group at 1031 cm^−1^ increased significantly. As the level of ultraviolet radiation increased, the peak intensity of C=O gradually increased, while the peak intensity of C=C gradually decreased. The change of C=O is because the asphalt molecular chain broke and formed free radicals under long-term ultraviolet irradiation, and then the formed free radicals combined with oxygen to produce several oxygen-containing groups. The absorption peak at 966 cm^−1^ is the C=C bending vibration absorption peak in SBS, and its peak intensity gradually decreases. This is because as the intensity of ultraviolet radiation increases, SBS absorbs ultraviolet light energy, and the molecular chain breaks. The absorption peaks of methyl and methylene at 1460 cm^−1^ and 1375 cm^−1^, respectively, increased slightly, and the amplitude of the absorption spectrum increased with the increase in ultraviolet radiation level. This is mainly due to the destruction of the C=C bond of the unsaturated olefin in the high-viscosity pitch under ultraviolet irradiation, forming a C-H bond. Compared with the original HVA, the amplitude of these two bands decreased slightly after UV aging, and with the increase in UV irradiance, the amplitude decreased more.

**Figure 4:**
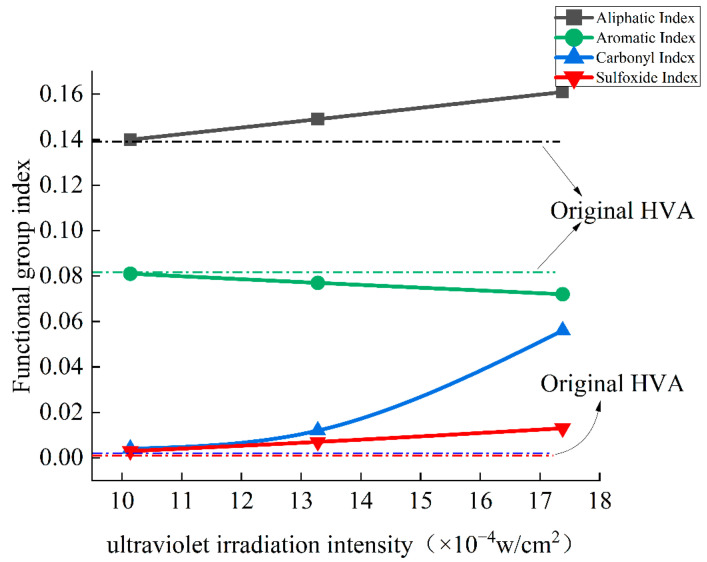
Variation of characteristic functional group index under different ultraviolet irradiation intensity.

As shown in Figure 4, when the ultraviolet irradiance reached the level of 10.14 × 10^−4^ w/cm^2^ under the three types of ultraviolet radiation intensity, the carbonyl group started to increase. When the ultraviolet irradiance increased to 13.28 × 10^−4^ w/cm^2^ and 17.38 × 10^−4^ w/cm^2^, the carbonyl index increased by two times and 17 times, respectively, indicating that with the increase in ultraviolet irradiance, ultraviolet rays have a strong promotion effect on the formation of carbonyl functional groups. The sulfoxide group vibration only appeared when the ultraviolet irradiance reached the level of 13.28 × 10^−4^ w/cm^2^, and when the ultraviolet irradiance increased to 17.38 × 10^−4^ w/cm^2^, the sulfoxide group index only increased by 85.7%. Compared with the carbonyl index, the growth rate was much smaller, indicating that for high-viscosity modified asphalt, the carbonyl functional group is more sensitive to ultraviolet rays, and the carbonyl index is used to evaluate the effect of ultraviolet aging more obviously.

### 3.2. The Effect of Temperature on Aging

**Figure 5:**
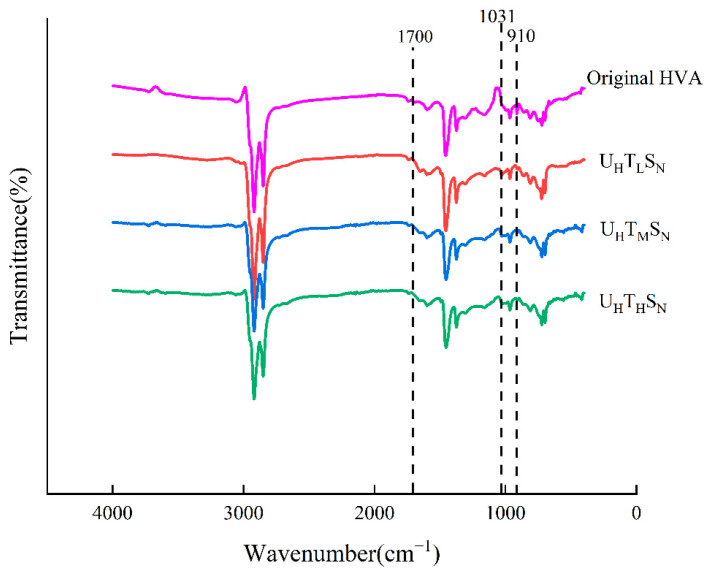
Variation of spectrum of different asphalt samples under different temperature levels.

It can be seen from Figure 5 that under different temperature levels, the absorption peak of sulfoxide group at 1031 cm^−1^ and the absorption peak of carbonyl group at 1700 cm^−1^ tended to strengthen. There was no new absorption peak during the aging process, indicating that the increase in temperature only led to the aging progresses, and no chemical reaction occurred to produce new substances. The peak of the polystyrene section near 910 cm^−1^ disappeared, which is due to the chemical reaction of the SBS polystyrene in the high-viscosity modified asphalt under thermal oxidative aging conditions [19].

**Figure 6:**
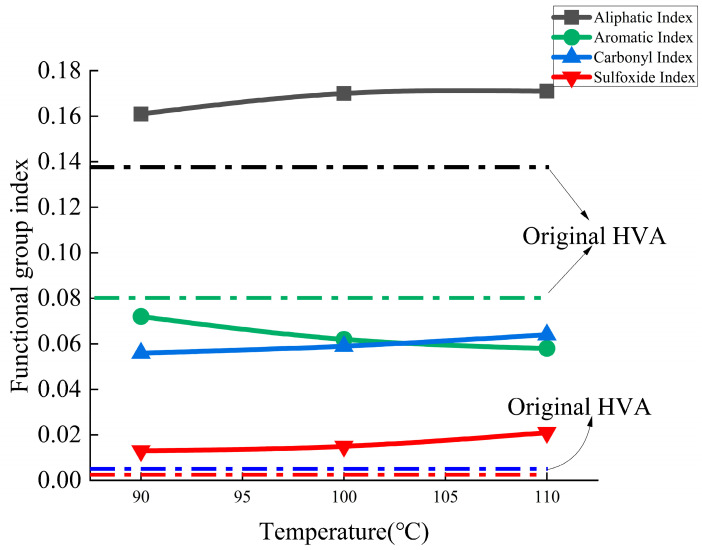
Change of functional group index under different temperature levels.

In terms of characteristic functional group index, as shown in Figure 6, as the temperature increased, the aging temperature increased from 90 °C to 110 °C, the aliphatic index increased by 6.21%, the aromatic index decreased by 19.4%, and the carbonyl index increased by 14.3%. The sulfoxide base index also increased by 61.5%. This showed that in the temperature range of 90–110 °C, the changes of aliphatic, aromatic, and carbonyl functional groups were not obvious. Unlike the sensitivity to ultraviolet rays, the sulfoxide group is more sensitive to temperature. When studying the thermal aging of high-viscosity modified asphalt, it is more appropriate to use the sulfoxide index as an evaluation index.

### 3.3. The Effect of Water Environment on Aging

**Figure 7:**
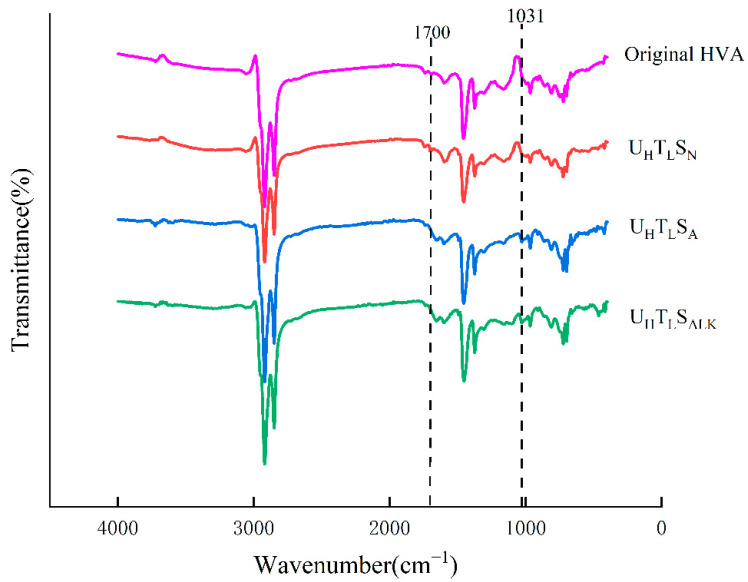
Variation of spectrum of different asphalt samples under different water environment.

Based on the combination of 17.38 × 10^−4^ w/cm^2^, 90 °C, and a neutral water environment, the water quality was changed, and the influence of saline-alkali water and acid rain on the aging behavior of high-viscosity modified asphalt was analyzed. It can be seen from Figure 7 that the infrared spectra of high-viscosity modified asphalt under different water quality did not show different absorption peaks, indicating that SO_4_^2−^ and NO_3_^−^ in acid rain solution, and Na^+^ and Cl^−^ in alkaline solution, did not chemically react with asphalt to generate new substances.

**Figure 8:**
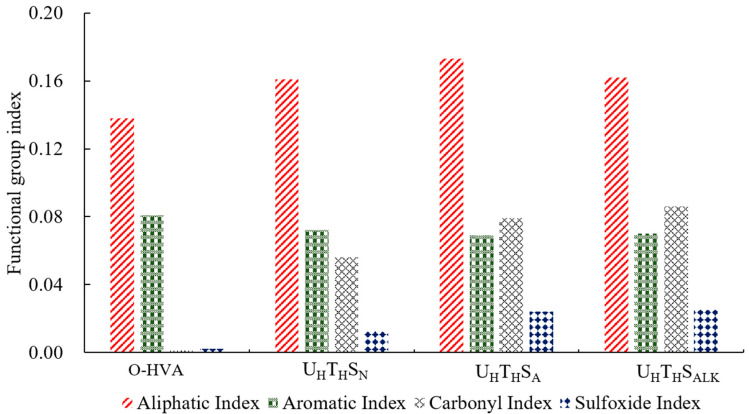
Change of characteristic functional group index under different water properties.

As shown in Figure 8, compared with neutral water, the aliphatic index of the high-viscosity modified asphalt in acid rain solution increased by 7.5%, the aromatic index decreased by 4.2%, the carbonyl index increased by 41.1%, and the sulfoxide group index increased by 84.6%. In the high-viscosity pitch in alkaline solution, the aliphatic index and the aromatic index hardly changed, the carbonyl index increased by 53.6%, and the sulfoxide group index increased by 92.3%. Under the action of acidic and alkaline solutions, the range of changes of aromatic phenol and saturated phenol was very small, indicating that the light and heavy components of high-viscosity modified asphalt have not changed due to the difference in water quality, but the carbonyl index and sulfoxide index had increased. From the changes of carbonyl and sulfoxide groups, acid rain and saline-alkali groundwater will increase the aging of high-viscosity modified asphalt. This may be due to the acidity of carboxylic acids and phenols in the asphalt. The substance dissolves and ionizes in the acid rain solution to a certain extent, and the neutralization reaction occurs in the alkaline solution, which accelerates the aging of the asphalt in the water, and the sodium hydroxide and asphaltic acid in the alkaline solution can react to form organic soap compounds.

### 3.4. Variance Analysis

The SPSS software was used to analyze the variance of the test results at the 95% confidence level, and the significance of the influence of different factors on the aging functional group index was compared. The results are shown in Table 5. Statistical analysis shows that UV intensity, temperature, and water quality conditions all have a significant impact on the functional group index. Among them, the degree of influence of ultraviolet intensity on the carbonyl index is greater than that of temperature and water quality environment, and the influence of temperature on the sulfoxide group index is greater than that of ultraviolet intensity and water quality environment. For the aliphatic index and the aromatic index, the three have the same significance.

### 3.5. Aging Kinetic Model of High-Viscosity Modified Asphalt

Asphalt binder meets the first-order reaction under conventional thermal and oxygen aging conditions [20], and its rate equation is:

(5) $$-\frac{dc}{dt}=kc$$

After integration:

(6) $$\int^{\;}-\frac{dc}{c}=\int^{\;}kdt$$

(7) −Inc=kt+B

When *t* = 0, *C = C*_0_, and *B* = −*Inc*_0_, so the general formula of first-order reaction kinetics can be obtained:

(8) $$Inc=Inc_{0}-kt$$

(9) $$In\left(c_{0}/c\right)=kt$$

Arrehenius put forward the concept of activation energy through a lot of research. He believes that molecules can react without contact if they have enough energy. These high-energy molecules are called activated molecules, and inactive molecules are transformed into activated molecules. The required energy is activation energy, as shown in the following formula:

(10) $$Ink=InA-\frac{E_{a}}{RT}$$

After obtaining the aging kinetic parameters and substituting it into Equation (10), we come to:

(11) $$\;In\left(\frac{c_{0}}{c}\right)=Ate^{-\left(E_{a}/R\right)/T}$$

where:

*E_a_*: activation energy;
*A*: Pre-reference factor;
*T*: Kelvin temperature;
*R*: Molar gas constant;
*c*: A functional group index;
*k*: Reaction rate constant at temperature *T*;
*t*: Aging time.

Take the high temperature and ultraviolet and acid rain solution environment combination (T + UV + S) as the aging condition, make the high-viscosity modified asphalt aged 0 h, 10 h, 20 h, 30 h, 40 h at the temperature level of 90 °C, 100 °C, 110 °C. The ultraviolet radiation level was 17.38 × 10^−4^ w/cm^2^, and the infrared spectrum scanning test was performed on the aged asphalt samples. The carbonyl index *I_C_* and the sulfoxide group index *Is* were used to establish the aging kinetic equation and calculate the kinetic parameters.

According to the Arrhrenius formula, the first-order aging kinetic model with *I_C_* and *I_S_* as parameters are obtained. The parameters are shown in Table 6.

(12) $$In\left(I_{c}/I_{c0}\right)=771.16te^{-3425.5/T}$$

(13) $$In\left(I_{s}/I_{s0}\right)=8.6te^{-1598.4/T}$$

**Figure 9:**
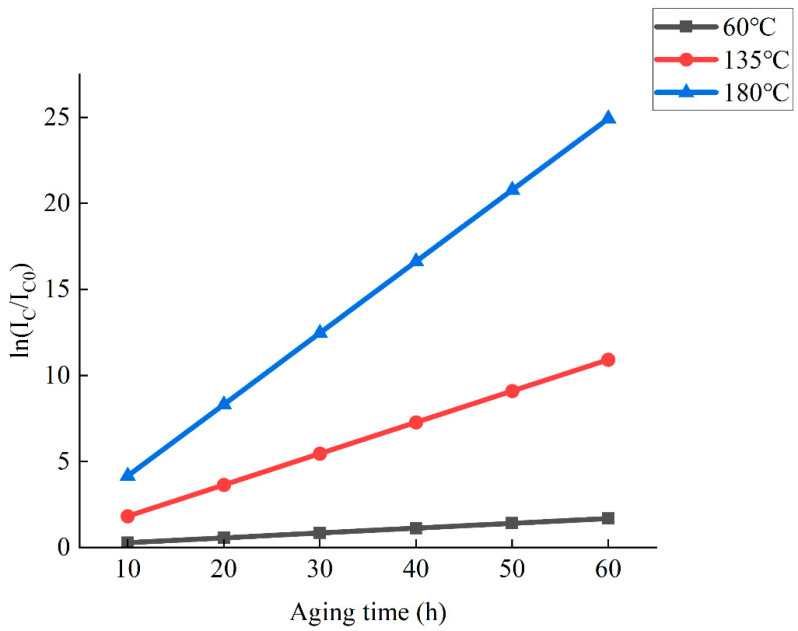
Changes of carbonyl functional groups at different temperatures.

Activation energy is an index used to evaluate the degree of difficulty of reaction. The greater the activation energy, the more difficult it is for asphalt molecules to be converted into activated molecules, the more difficult it is for the reaction to generate new free radicals, and the better the anti-aging performance. Table 6 shows that activation energy has a greater impact on the reaction rate and temperature. For a reaction with large activation energy, temperature has a greater impact on the reaction rate. Therefore, the activation energy also determines the sensitivity of the reaction rate to temperature. Compared with *I_S_*, the aging kinetic model established with *I_C_* as the parameter has greater activation energy. The greater the influence of temperature on the aging reaction rate, the more suitable the established model is for traditional indoor simulation of thermal oxygen aging. As Figure 9 shows, this model can be used to predict the changes of carbonyl functional groups of high-viscosity modified asphalt under different temperature environments, and provide a theoretical basis and reference for reducing the performance of high-viscosity modified asphalt due to thermal-oxidative aging during actual production, transportation, and construction.

## 4. Conclusions

This study investigated the aging behaviors of high-viscosity asphalt subjected to complex aging conditions based on the FTIR technique, and the results demonstrated that the high-viscosity asphalt was sensitive to the combined aging factors by the change of chemical functional groups. There are some main conclusions summarized as below:

(1) Compared with the unaged high-viscosity modified asphalt, the increase in UV irradiance, the increase in temperature and the change of water quality cannot make the high-viscosity modified asphalt generate new functional groups. Under the three environmental factors, the high-viscosity modified asphalt has no chemical reaction, only physical changes.
(2) When studying ultraviolet aging, it is more obvious to use the carbonyl index as the evaluation index, while the sulfoxide group is more sensitive to temperature, and it is more appropriate to use the sulfoxide index as the heat aging evaluation index.
(3) The aging kinetic model established with the carbonyl index as a parameter has greater activation energy, and the established model is more suitable for traditional indoor simulation of thermal-oxidative aging.

## Figures and Tables

**Table 1:** Basic properties of two asphalt samples.

Asphalt Types	Penetration 25 °C (0.1 mm)	Softening Point (°C)	5 °C Ductility (cm)	60 °C Dynamic Viscosity (Pa·s)
SBS	64	71.2	36.5	5015
HVA	44.3	86.4	45.3	73,527

**Table 2:** Technical specification of TPS high-viscosity agent.

Project	Unit	Results	Standard
Particle size	mm	4.2	JT/T 860.2
Density	g/cm^3^	0.8	GB/T1033
Absorption	%	0.6	-

**Table 3:** Aging program.

Asphalt Specimen	UV Irradiance (w/cm^2)^	Temperature (°C)	Water Environment
O-HVA	/	/	/
U_L_T_L_S_N_	10.14 × 10^−4^	90	neutral
U_M_T_L_S_N_	13.28 × 10^−4^	90	neutral
U_H_T_L_S_N_	17.38 × 10^−4^	90	neutral
U_H_T_M_S_N_	17.38 × 10^−4^	100	neutral
U_H_T_H_S_N_	17.38 × 10^−4^	110	neutral
U_H_T_L_S_A_	17.38 × 10^−4^	90	acidic
U_H_T_L_S_ALK_	17.38 × 10^−4^	90	alkaline

**Table 4:** Infrared spectrum analysis of asphalt.

Wavenumber (cm^−1^)	Spectral Peak Attribution
2924	Asymmetric stretching vibration of C-H in methylene
2852	Symmetrical stretching vibration of C-H in methylene
2729	Stretching vibration of aldehyde group
1686	C=O stretching vibration
1671	C=O stretching vibration of primary amide carbonyl
1600	Respiratory vibration of asymmetrically substituted benzene ring
1461	Scissor vibration of methylene (—CH_2_—)
1377	Umbrella vibration of methyl (—CH_3_—)
1031	Stretching vibration of sulfoxide group (S=O)
812\868	Stretching vibration of benzene ring
747	Bending vibration of aromatic branched chain
722	Synergistic vibration of methylene segment (CH_2_) n (n ≥ 4)

**Table 5:** Analysis of variance results.

Project	Deviation Sum of Squares	Degree of Freedom	Mean Square Error	F	*p*
(1) Aliphatic index					
UV intensity	2.16 × 10^−4^	3	2.16 × 10^−4^	2.892	0.012
Temperature	1.29 × 10^−3^	3	1.29 × 10^−3^	55.099	0.011
Water environment	1.12 × 10^−3^	3	1.12 × 10^−3^	53.556	0.012
(2) Aromatic Index					
UV intensity	2.81 × 10^−5^	3	2.81 × 10^−5^	2.770	0.013
Temperature	4.33 × 10^−4^	3	4.33 × 10^−4^	16.673	0.015
Water environment	1.70 × 10^−4^	3	1.70 × 10^−4^	46.286	0.011
(3) Carbonyl Index					
UV intensity	7.26 × 10^−4^	3	7.26 × 10^−4^	3.967	0.004
Temperature	4.98 × 10^−3^	3	4.98 × 10^−3^	61.796	0.013
Water environment	7.70 × 10^−3^	3	7.70 × 10^−3^	62.551	0.013
(4) Sulfoxide Index					
UV intensity	6.01 × 10^−5^	3	6.01 × 10^−5^	31.852	0.025
Temperature	4.86 × 10^−4^	3	4.86 × 10^−4^	15.429	0.007
Water environment	5.80 × 10^−4^	3	5.80 × 10^−4^	26.173	0.012

**Table 6:** Arrehenius model parameters.

Kinetic Parameters	Aging Temperature (°C)	*k*	*A*	*E_a_* (kJ·mol)
*I_c_*	90	0.0650	771.16	28.47
	100	0.0795		
	110	0.1010		
*I_s_*	90	0.1095	8.600	13.28
	100	0.1156		
	110	0.1342		

## Data Availability

All the original data of this article can be obtained by contacting the corresponding author.
